# Knowledge and attitudes about dementia among nursing students in Vietnam: a cross sectional study

**DOI:** 10.21203/rs.3.rs-4586709/v1

**Published:** 2024-07-15

**Authors:** Dung Thi My Pham, An Dang Do, Hien Thi Thu Do, Anh Ngoc Nguyen, Binh Thanh Nguyen, Mai Do

**Affiliations:** Hanoi Medical College; The University of Tokyo; Hai Duong Medical Technical University; National Geriatric Hospital; National Geriatric Hospital; Tulane University School of Public Health and Tropical Medicine

**Keywords:** dementia, Alzheimer’s disease, knowledge, attitude, nursing, education, mental health, aging

## Abstract

**Introduction:**

Dementia is one of the most pressing health concerns in ageing population, posing significant burdens not only on the well-being and independence of people with dementia (PWD), but also on their families and communities. Building capacity for nursing students is essential for effectively enhancing the quality of life for affected people. However, various studies have highlighted knowledge gaps concerning dementia among nursing students worldwide. This study aimed to examine knowledge and attitude preparedness for dementia care among nursing students in Vietnam and associated factors.

**Methods:**

We used cross-sectional study design with four medical schools in northern, central and southern Vietnam which were selected based on convenience. Participants included full-time diploma senior nursing students (N = 600). A self-administered questionnaire consisted of 34 true-false questions evaluating students’ knowledge about dementia (ADKS) and 20 items using a 5-point Likert scale assessing their attitudes toward dementia (DAS). The tools were piloted and validated with 60 students with good internal reliability.

**Results:**

Nursing students exhibited insufficient knowledge of dementia but displayed positive attitudes towards dementia. Engaging four-year diploma (β = 0.501), being female (β = 0.827), and achieving greater academic performance (β = 0.795) statistically influenced nursing students’ knowledge of dementia (p < 0.05). Similarly, being female (β = 2.179), and possessing better knowledge of dementia (β = 2.740) statistically effected nursing students’ attitude toward dementia (p < 0.05).

**Conclusions:**

Students with greater academic achievement and females demonstrated better knowledge and attitudes toward dementia. To better nursing students’ preparedness for dementia care, education on dementia care, especially practical training should be paid more attention.

## 1. Introduction

By 2050, the population aged 60 and older is projected to constitute approximately 20% of the global population, with most of the increase occurring in low- and middle- income countries (LMICs) (United Nations Population Fund, 2012; WHO, 2023). Defined as a term for different diseases that impair people’s memory, cognition and daily activities, dementia affects an estimated 55 million individuals worldwide, with over 60% residing in LMICs according to the World Health Organization (WHO) (WHO, 2023.). Dementia exhausts PWD’s health, socialization and economy. In 2015, the estimated global cost for dementia was up to 818 billion USD (~ 1.1% of global gross domestic product - GDP) (WHO, 2023), and an increase of approximately 500 billion US dollars in expenses for dementia was estimated between 2015 and 2019 (United Nations Population Fund, 2012; WHO, 2023.).

Currently, a cure is unavailable for dementia, although several medicines and therapies help to prevent, slow down or lessen the negative impacts of dementia on affected people. To enable nurses who are among the key professionals providing care helping improve the quality of life of PWD, it is necessary to equip nursing students with proper knowledge and attitudes. Nevertheless, different studies revealed knowledge gaps in dementia care among nursing students globally [4]. Poor knowledge and positive/moderate attitudes of dementia were found among Indian nursing students (2015) [5], Jordanian nursing students (2022) [6], Turkish nursing students (2023) [7], Indonesian nursing students (2020) [8], and in Tasmanian nursing students (2025) [9]. We found only one study concluded adequate knowledge and positive attitudes of dementia in Maltese nursing students (2013) [10]. Such gaps can have adverse effects on the health and wellbeing of PWD.

Since 2011, Vietnam has been an aging nation and is currently among the countries aging the fastest [11]. According to Nguyen’s estimation, about 7.9% of Vietnamese people who age 60 and above are living with dementia [12]. The average longevity of Vietnamese poeple (75.6 years) ranks at 2nd in Asian and 56th globally [11], which means that those with dementia can potentially have a long life. The elderly population currently accounts for approximately 10% of the national population and is expected to be on the rapid rise in the coming time [11]. It is critical that health and support systems exist to ensure that these elders can enjoy a long and quality life. Nevertheless, anecdotes suggest that health systems, including personnel, are ill equipped to provide care and support to this fast-growing population. Dementia care remains an afterthought, lacking strategic preparedness planning to meet the increasing demands of resources. This study aimed to assess knowledge and attitudes about dementia and dementia care among nursing students, and to identify factors related to such knowledge and attitudes.

## 2. Methods

### 2.1. Study design

A cross-sectional survey was employed with full-time diploma nursing students in their last year. Four medical schools providing nursing program in Vietnam were selected for this study, including Hanoi Medical College, Hai Duong Medical Technical University (located in the North), Vinh Medical University (in the Central), Ho Chi Minh University of Medicine and Pharmacy (in the South). These public schools, which are well-known in educating medical personnel were selected because they train the biggest number of medical students in their locals, including nursing students. Of which, Hanoi school educates 3-year diploma nursing students, 4-year nursing students are educated in the remaining schools. Comparing schools with 3- versus 4-year program of training may allow some insight to the effectiveness of these programs in equipping students’ knowledge and attitudes of dementia. Ethical approval was provided by the Research Ethics Committee of Hanoi Medical University.

In total, 600 students were recruited for the study. Students who had completed the subject related to ageing care (including dementia care), accessible without participating in the pilot survey were eligible. Random proportionate sampling was used to select the research subjects.

### 2.2. Data collection

A self-administrated questionnaire consisting of 34 true/false questions evaluating nursing students’ knowledge of dementia, and 20 items assessing their dementia attitudes was developed on Google forms and online delivered to participants. Even though informed consent forms had been attained before the link was sent by the research team members who work in the participating schools, students were asked to choose the yes option to confirm their voluntary participation. The questionnaire only became available to those who confirmed their willingness to participate.

### 2.3. Instruments development and validation

#### * Instruments development:

##### Dementia knowledge instrument:

Questions assessing dementia knowledge were developed primarily based on the Alzheimer’s Disease Knowledge Scale (ADKS) of Carpenter et al [13], we excluded several items that may not culturally fit Vietnamese setting, for instance: Most people with AD live in nursing homes, AD is a type of dementia etc. Additionally, several questions were adapted from the research tools assessing dementia knowledge in Vietnamese Americans of Lee et al. [14], and Nguyen et al. [15]. Examples of these additional questions included: AD is a form of insanity, A person will almost certainly get AD if they just live long enough, Dementia shortens life expectancy after onset. In consultation with a medical doctor and the chief of nurses who were working in the Neurology and Alzheimer Disease – National Geriatric Hospital, together with cultural consideration, an ADKS consisting of 35 true/false items were developed and adapted for cultural relevance. A participant’s score of dementia knowledge was calculated based on the number of correct responses (each correct answer was assigned on point); the higher the score, the better knowledge a student possessed.

##### Dementia attitudes instrument:

Dementia Attitudes Scale (DAS) developed by O’Connor et al.[16] was used to evaluate nursing students’ attitudes towards dementia. Some items were excluded, such as: I feel uncomfortable being around people with ADRD, I feel relaxed around people with ADRD ... as these items were somehow overlap with the selected item (I am comfortable touching people with ADRD). We also added some items evaluating participants’ attitudes related to popular beliefs in Vietnam, such as: Some people believe that doing bad things in previous lives or in early ages could result in their suffering from dementia in their later lives, People with dementia should be taken care of at home by their families rather than in nursing homes. Finally, a DAS consisting of 20 items with 5-point Likert scale (strongly disagree, disagree, neutral, agree and disagree) was developed. An attitude score was calculated by summing up the score of each item; the higher the score, the more positive attitude a student had towards dementia and PWD.

#### * Instruments validation:

Before fieldwork, a pre-test was implemented on 10% of the sample size (60 students, including 30 three-year diploma and 30 four-year diploma nursing students of the surveyed schools). A self-administrated questionnaire consisting of both ADKS and DAS was accessible to these students.

For the ADKS [12], one item (Dementia is infectious) was removed from the instrument since it did not contribute to the measure of dementia knowledge. By removing that item from the instrument, internal reliability (Cronbach’s alpha) was increased from 0.68 to 0.69. For the DAS, Cronbach’s alpha was 0.88. Therefore, all the items were retained for the survey. The complete lists of items for ADKS and DAS were presented in Appendice 1.

### 2.4. Sample size

The formula for cross-sectional study with prevalence (P) was used. With α = 0.05, confident limit around the point estimate (d) = 0.05, P = 0.531 (we used the rate of South Korean nursing students having correct knowledge about dementia since no similar statistics were available in Vietnam) [17], and the design effect = 1.5, the calculated sample size was 574. Totally, 600 nursing students were recruited for the survey. Since the quantity of 3-year diploma students and the number of 4-year diploma students who met the selection criteria were quite equally, our sample consisted precisely a half of 3-year diploma students and a half of 4-year diploma students.

### 2.5. Data analysis

Data was analyzed with Stata MP Version 17. Descriptive statistic was used to calculate the frequency, percentage, dementia knowledge and attitudes’ mean, median, standard deviation, max, min. Scale analysis was applied to examine the reliability of the survey instruments. Linear regression were used to examine the correlation between respondents’ general characteristics and their knowledge and attitudes about dementia, and the interactions between independent variables in the models.

### 2.6. Ethics in research:

The research was implemented after receiving written approval from the National Council for Ethics in Biomedical Research (Hanoi Medical University) and written approval from the participating schools. Informed consent forms were obtained from all participants. Personal information was not collected. Participants were paid an allowance equal to 2 USD for each questionnaire survey respondent, 3 USD for each FGD participant, and 40 USD for each key informant, according to approved cost-norms and transparently presented in the informed consent.

## 3. Results

### 3.1. Demographic characteristics

The demographic characteristics of the participants are described in Table 1. The participants were recruited from Hanoi Medical College (HMC) (300, 50.0%), Hai Duong Medical Technical University (HMTU) (103, 17.2%), Vinh Medical University (VMU) (88, 14.7%) and Ho Chi Minh University of Medicine and Pharmacy (UMP) (109, 18.2%). The majority of participants were female (88.5%), from rural areas (63.5%), had good learning performance (62.8%) (the learning results of students were classified hierarchically into five categories, including: excellent, very good, good, average, and poor/very poor corresponding to the A, B, C, D and F scales), and were inexperienced in dementia care (71.7%). Their ages ranged from 19 to 30 years (mean ± SD: 20.8 ± 1.14).

### 3.2. Knowledge and attitudes toward dementia

Table 2 indicates that dementia knowledge among nursing students left much room for improvement, with almost all of the knowledge domains having at least one item receiving less than half of the number of correct answers, except for the life impacts domain. In particular, approximately 4/5 of the students wrongly assumed that using reminder notes helps decrease AD (M ± SD: 0.15 ± 0.36), reminding people with AD that they are repeating things is useful (M ± SD: 0.25 ± 0.43), and caregiving is immediately required as soon as individuals with AD start struggling with self-care (M ± SD: 0.14 ± 0.35). Nursing students illustrated poor knowledge about AD progression (3.38 ± 0.98, out of 5), Assessment and diagnosis (2.53 ± 1.09, out of 6), Treatment & management (3.26 ± 0.90, out of 5); Caregiving (1.19 ± 0.74, out of 3); and inadequate knowledge about Risk factors (4.03 ± 1.10, out of 6), and Symptoms (4.15 ± 1.10, out of 6). Generally, the mean total knowledge score (20.82 ± 2.96, out of 34) indicated their inadequate knowledge about AD.

The students’ attitudes of dementia are described in Table 3. The mean scores in attitudes range from 3.12 to 3.94 (out of 5). Even though overall mean attitude score (65.54 ± 8.25, out of 90) demonstrated that participants held positive attitudes towards dementia, their attitudes toward some items were relatively limited, with only 231/600 (38.5%) participants agreeing that working with people with ADRD is beneficial, and 242/600 (40.3%) reporting that they feel comfortable touching people with ADRD, and 133 (22.2%) stating that PWD becomes a burden to their families and society.

### 3.3. Factors associated with dementia knowledge and attitudes

The results of the multivariable linear regression analysis shown in Table 4 indicate that having a 4-year diploma (β = 0.501), being female (β = 0.827), and being a good student (β = 0.795) were statistically positively associated with nursing students’ knowledge of dementia (p < 0.05). No statistical correlation was found between their living place or experience in dementia care and their knowledge of dementia (p > 0.05).

In addition, females (β = 2.179), and knowledge about dementia (β = 2.740) were statistically positively associated with nursingstudents’ attitude towards dementia (p < 0.05) (Table 5). Statistically insignifi cant correlation was found between the type ofdiploma, place of residence, learning performance, or experience in dementia care and attitudes toward dementia (p > 0.05).

Moreover, no interaction was found between independent variables in the two linear regression models.

## 4. Discussion

In this study, we found that nursing students who had completed dementia care training, were predominantly female, from rural areas, and inexperienced in dementia care exhibited inadequate knowledge about AD, with notable gaps in understanding its progression, assessment, treatment, and caregiving. Similar findings were published in previous studies by Scerri, Poreddi, Eccleston, Aslan, Aljezawi, Evripidou, and Sunaryo [4–10]. In this study, their knowledge gap was partially explained by not only the limited amount of time for their theoretical training (within 1 to 5 learning hours, varied between schools) on dementia, but was also rationalized by their inexperience in dementia care as well. In one study in 2016 conducted by Kimzey, nursing students who received clinical experience in AD care had improved knowledge about AD, compared to those who engaged only in an online module or those who received no dementia-specific intervention [18]. Other researches have suggested that evidence-based educational interventions for dementia care, and proper clinical placement could significantly strengthen nursing students’ preparedness in dementia care [4, 9, 19].

The findings also showed that even though the overall attitude toward dementia was positive, which is consistent with previous findings of Scerri, Poreddi, Aslan, and Sunaryo [5–8, 10], some areas still need attention. For example, only slightly more than one-third of the participants agreed that working with people with ADRD was beneficial. This item was also among the items received most negative attitude among the DAS items in the research of Sunaryo in Indonesian nursing students (2020) [8], in the research of Poreddi on Indian nursing students (2015) [5], and in the research of Aljezawi on Jordanian nursing students (2022) [6]. This perception may be a result of the inefficiency of healthcare in lessening memory loss and cognitive impairment for PWD. Furthermore, up to 22.2% of the respondents stated that PWD become a burden to their families and society. In Vietnamese setting, the majority of elderly individuals, including PWD are mainly cared for and financially supported by their offspring without the pension and affordability of nursing homes/paid care services. Therefore, some elders with chronic diseases (including dementia) become a financial and caring burden for their families, especially low- and middle-income families. Moreover, more than half of the nursing students (40.3%) reported that they did not feel comfortable touching people with ADRD, which also reflects their inadequate knowledge and inexperience in dementia care.

This study highlighted the role of gender (female students) and time length of nursing education which were also confirmed by previous studies by Aslan in 2023 [7], Scerri in 2013 [10], and Laura in 2022 [20]. Engaging 4-year diploma, being female, and achieving good academic performance were statistically positively associated with their knowledge of dementia. In this study, we also found that educational level was not statistically correlated with dementia knowledge. This finding is similar to those of several previous studies in LMICs. For example, in Khait’ findings in Jordan in 2021, students’ gender, current grade point average (GPA), and education level statistically effected the total mean dementia knowledge score of nursing students [21]. Meanwhile, statistical indifference in knowledge between academic levels was found by Aljezawi in Jordanian nursing students (2022) [6]. We did not observe a statistical correlation between the residents of nursing students or their experience in dementia care and their knowledge of dementia (p > 0.05). It is opposite to study by Aslan, which found that those who were living in a city center (p < 0.05) having statistically higher total score in dementia knowledge [7]. Additionally, a statistically positive correlation between students’ knowledge and their previous experience in dementia care during their clinical placement was found by Scerri in Malta [10], by Laura in Spain (2022) [20], and by Sunaryo in Indonesia [8], but students’ knowledge was not statistically associated with their experience in caring for relatives with ADRD (p > 0.05) [20]. It is possible that their limited experience was not sufficient to result in a statistical difference in their knowledge about dementia.

Similarly, being female and possessing knowledge about dementia were statistically positively associated with their attitude towards dementia. Being female was statistical correlated with attitude towards dementia (p < 0.05) which was also found by Aslan in Turkey [7]. Females are generally seen as sensitive, gentle and more suitable for jobs that require patience, hard work, and paying attention to details such as nursing or dementia care. This personality discrepancy may partly explains the more positive attitudes about dementia among female students. In addition, better knowledge about dementia statistically impacted students’ attitude of dementia (p < 0.05). A similar association was illustrated by Aslan (2023) (p < 0.05) [7], and Kwon (2017) (p < 0.05) [17]. This understandable association between knowledge and attitude could be explained by the fact that better awareness of dementia leads to more confidence in dementia care, then leads to increased comfort and positive perceptions in interacting with/working with PWD. Kimzey (2016) presented evidence of more positive attitudes about people with AD in nursing students who received clinical experience in AD care than those who participated in online module or those who received no dementia intervention [18]. Remarkably, while no statistical correlation was found between students’ experience in dementia care and their attitude towards dementia in this research (p > 0.05), the opposite result was found by Aslan (2023) [7, 22], and Scerri (2013) (p < 0.05) [10]. Moreover, a statistically insignificant correlation was found between students’ attitude of dementia and their type of diploma, living place, and learning performance (p > 0.05). However, high heterogeneity was found among relevant studies. For instance, Aslan (2023) reported a statistically insignificant association between nursing students’ academic year and their attitude of dementia (p > 0.05), but statistically positive correlation between their living place and their attitude towards dementia (p < 0.05) [7]. Similarly, a statistically insignificant difference in students’ attitude by their academic levels was found by Aljezawi (2022) in Jordan (p > 0.05) [6]. Meanwhile, Scerri found out that academic year was statistically associated with positive attitudes in Malta (2013) (p < 0.05) [10]. These discrepancies may resulted from from differences in the research subjects and sample sizes.

### Limitations

A few limitations are worth mentioning. The study was conducted in 4 public schools, therefore, the results may not be generalizable to private schools, which also educate nursing students. An increasing numbers of private schools together with dozens of existing schools that were established by individuals outside the government system or by multi-sector companies/corporations are educating thousands of health professionals each year in Vietnam. Moreover, the research was carried out with full-time nursing students, meanwhile, Vietnamese medical schools also provide informal nursing education to unemployed and/or employed students who attend classes in the evening and/or on weekends (i.e., similar to on-the-job training), and to those who have already possessed another degree/certificate (degree 2), therefore, the results of this study may not be generalizable to all nursing students. Finally, statistically significant associations in students’ knowledge and/or attitude of dementia and their family history and living status were found in previous studies [7, 10], but that information was not collected in our survey, limiting our analyses.

## 5. Conclusions

Nursing students were inadequately prepared in knowledge about dementia, especially in AD assessment and diagnosis, treatment & management; and caregiving. Despite their overall positive attitudes towards dementia, their attitudes towards some items remained relatively limited. Students with higher academic achievement and who were female demonstrated better knowledge and attitudes of dementia. The key recommendations from the study are: promoting both theoretical and practical training on dementia care for nursing students, of which, more attention should be given to AD caregiving, assessment and diagnosis, treatment & management. In clinical placement, nursing students should be provided more opportunities to practice with PWD in order to not only strengthen their knowledge, but also enhance their dementia attitudes.

## Figures and Tables

**Table 1:** Demographic characteristics of participants (n = 600)

Characteristic	Categories	Frequency	Percent
School (type of diploma)	Hanoi Medical College (3-year)	300	50.0
	Hai Duong Medical Technical University (4-year)	103	17.2
	Vinh Medical University (4-year)	88	14.7
	Ho Chi Minh University of Medicine and Pharmacy (4-year)	109	18.2
Age (M ± SD)		20.80 ± 1.14	
Sex	Other	69	11.5
	Female	531	88.5
Residential area	Urban	219	36.5
	Rural	381	63.5
Learning result	Average/Poor	82	13.7
	Good	377	62.8
	Excellent/very good	141	23.5
Experience in dementia care	No/not remember	430	71.7
	Yes	170	28.3
Relationship with PWD you took cared (n = 170)	Family members/ relatives/ acquaintances	35	20.6
	Patients at hospitals	126	74.1
	Both relatives and patients	9	5.3

PWD: People living with dementia; SD: Standard Deviance

**Table 2:** Students’ knowledge about dementia

	Correct answer n (%)
AD is a form of insanity. (F)	39.3 (236)
A person will almost certainly get AD if they just live long enough. (F)	69.3 (416)
An older man is more likely to develop AD than an older woman. (F)	56.8 (341)
Genes can only partially account for the development of AD. (T)	81.3 (488)
When the brain is deprived of oxygen, nutrition or when the neurotransmitters are impaired, dementia can appear. (T)	88.0 (528)
Consuming fatty fish (rich in DHA) or omega-3 polyunsaturated fatty acids may reduce the risk of dementia. (T)	68.0 (408)
**Rate of students answered correctly all the 6 questions about AD’s Risk factors** **M ± SD**	**8.8 (53)** **4.03 ± 1.10**
Trouble handling money or paying bills is a common early symptom of AD. (T)	84.8 (509)
Tremor or shaking of the hands or arms is a common symptom in people with AD. (F)	42.0 (252)
Stuttering is an inevitable part of AD. (T)	92.5 (555)
One symptom that can occur with AD is believed that other people are stealing one’s things. (T)	55.7 (334)
Most people with AD remember recent events better than things that happened in the past. (F)	49.2 (295)
Difficulty in remembering the content of a conversation or not fully understanding the meaning of words heard are early symptoms of dementia. (T)	91.0 (546)
**Rate of students answered correctly all the 6 questions about AD’s Symptoms M ± SD**	**10.5 (63)** **4.15 ± 1.10**
After symptoms of AD appear, the average life expectancy is 6– 12 years. (T)	61.2 (367)
Dementia shortens life expectancy after onset. (T)	62.7 (376)
A person with AD becomes increasingly likely to fall down as the disease gets worse. (T)	78.5 (471)
Eventually, a person with AD will need 24-hr supervision. (T)	92.5 (555)
In rare cases, people have recovered from AD. (F)	43.3 (260)
**Rate of students answered correctly all the 5 questions about AD’s Course** **M ± SD**	**11.3 (68)** **3.38 ± 0.98**
It is safe for people with AD to drive, as long as they have a companion in the car at all times. (F)	54.7 (328)
People with AD are particularly prone to depression. (T)	83.0 (498)
A person who has AD will experience both mental and physical decline. (T)	90.7 (544)
**Rate of students answered correctly all the 3 questions about AD’s Life impact** **M ± SD**	**45.0 (270)** **2.28 ± 0.76**
If trouble with memory and confused thinking appears suddenly, it is likely due to AD. (T)	27.0 (162)
Symptoms of severe depression can be mistaken for symptoms of AD. (T)	75.2 (451)
Among persons over age 75, forgetfulness most likely indicates beginning of AD. (F)	34.2 (205)
Senescence forgetfulness progresses with advancing age, resulting in patients being unable to recognize their families. (F)	15.5 (93)
Medicine taken for high blood pressure can cause symptoms looking like AD. (T)	42.2 (253)
AD can be diagnosed by a blood test. (F)	58.8 (353)
**Rate of students answered correctly all the 6 questions about AD’s Assessment and diagnosis** **M ± SD**	**0.2 (1)** **2.53 ± 1.09**
People whose AD is not yet severe can benefit from psychotherapy for depression and anxiety. (T)	84.3 (506)
When a person has AD, using reminder notes is a crutch that can contribute to decline. (F)	15.3 (92)
There is no cure for AD at present. (T)	86.5 (519)
Medications are available to help control behavioral symptoms of AD. (T)	61 (366)
Medications are available to delay progression of AD. (T)	78.7 (472)
**Rate of students answered correctly all the 5 questions about AD’s Treatment and management** **M ± SD**	**4.2 (25)** **3.26 ± 0.90**
When people with AD repeat the same question or story several times, it is helpful to remind them that they are repeating themselves. (F)	25 (150)
If a person with AD becomes alert and agitated at night, a good strategy is to try to make sure that the person gets plenty of physical activity during the day. (T)	79.7 (478)
When people with AD begin to have difficulty taking care of themselves, caregivers should take over right away. (F)	14.3 (86)
**Rate of students answered correctly all the 3 questions about AD’s Caregiving** **M ± SD**	**4.8 (29)** **1.19 ± 0.74**
**Total ADKS (34)**	**20.82 ± 2.96**

AD: Alzheimer disease; T-True is the correct answer; F-False is the correct answer. ADKS: Alzheimer’s Disease Knowledge Scale

**Table 3:** Students’ attitudes about dementia (n = 600)

	Item	Students’ attitudes n (%)		
		Disagree	Neutral	Agree
1	Working with people who have Alzheimer disease and related disorders (ADRD) is beneficial to themselves, their families and communities.	155 (25.8)	214 (35.7)	231 (38.5)
2	I am afraid of people with ADRD.*	358 (59.7)	172 (28.7)	70 (11.7)
3	People with ADRD can be creative.	114 (19.0)	177 (29.5)	309 (51.5)
4	I am comfortable touching people with ADRD.	62 (10.3)	296 (49.3)	242 (40.3)
5	Every person with ADRD has different needs that should be taken care of.	44 (7.3)	95 (15.8)	461 (76.8)
6	I would avoid an agitated person with ADRD. *	367 (61.2)	165 (27.5)	68 (11.3)
7	It is important to know the past history of people with ADRD.	39 (6.5)	85 (14.2)	476 (79.3)
8	It is possible to enjoy interacting with people with ADRD.	48 (8.0)	195 (32.5)	357 (59.5)
9	People with ADRD should still be supported to enjoy life.	23 (3.8)	116 (19.3)	461 (76.8)
10	I admire the coping skills of people with ADRD.	32 (5.3)	146 (24.3)	422 (70.3)
11	People living with ADRD are among those who should be prioritized to be support in our society.	25 (4.2)	117 (19.5)	458 (76.3)
12	Protecting reputation and dignity of people living with ADRD is the responsibility of surrounding people.	26 (4.3)	95 (15.8)	479 (79.8)
13	People living with ADRD could still be beneficial to our society.	41 (6.8)	191 (31.8)	368 (61.3)
14	There are many things we can do to improve living quality of people with dementia.	24 (4.0)	93 (15.5)	483 (80.5)
15	Some people believe that doing bad things in previous lives or in early ages could result in their suffering from ADRD in their later lives.*	381 (63.5)	120 (20.0)	99 (16.5)
16	People with dementia become a burden to their families and society. *	223 (37.2)	244 (40.7)	133 (22.2)
17	In case of meeting a person living with ADRD, most people are caring or willing to help her/him.	44 (7.3)	195 (32.5)	361 (60.2)
18	If someone misbehaves with a person with ADRD, she/he may be condemned by surrounding people.	67 (11.2)	143 (23.8)	390 (65.0)
19	People with dementia should be taken care of by their families at home rather than going to nursing homes. (−)	147 (24.5)	249 (41.5)	204 (34.0)
20	In case of my family member living with dementia, I would take care of him/her at home rather than getting him/her to a nursing house.(−)s	89 (14.8)	223 (37.2)	288 (48.0)
	**Total DAS (18)**	**65.54 ± 8.25**		

* reverse scored items; (−) non scored items; ADRD: Alzheimer disease and related disorders; DAS: Dementia attitude scale

**Table 4:** Factors correlated with dementia knowledge (n = 600)

	Bivariate linear regression					Multiple linear regression				
	Unstandardized Coefficients		Standardized Coefficients	t	Sig.	Unstandardized Coefficients		Standardized Coefficients	t	Sig.
	B	SE	Beta			B	SE	Beta		
(Constant)						19.093	.482		39.620	.000
Type of diploma	.543	.241	.092	2.257	.024	.501	.246	.085	2.035	.042
Sex	.961	.377	.104	2.551	.011	.827	.377	.089	2.192	.029
Residential area	.172	.251	.028	.686	.493	− .008	.254	− .001	− .030	.976
Learning result	.853	.350	.099	2.436	.015	.795	.351	.092	2.263	.024
Experience in dementia care	.356	.268	.054	1.327	.185	.222	.269	.034	.829	.408

**Table 5.** Factors correlated with dementia attitude (n=600)

	Bivariate linear regression					Multiple linear regression				
	Unstandardized Coefficients		Standardized Coefficients	t	p	Unstandardized Coefficients		Standardized Coefficients	t	p
	B	SE	Beta			B	SE	Beta		
(Constant)						62.176	1.341		46.357	.000
Type of diploma	.967	.673	.059	1.436	.151	.580	.686	.035	.846	.398
Sex	2.612	1.051	.101	2.484	.013	2.179	1.050	.084	2.076	.038
Residential area	.079	.700	.005	.113	.910	.228	.706	.013	.324	.746
Learning result	.347	.981	.014	.353	.724	−.158	.978	−.007	−.162	.871
Experience in dementia care	.678	.748	.037	.907	.365	.441	.745	.024	.591	.555
Knowledge of dementia	2.918	.680	.173	4.293	.000	2.740	.689	.162	3.978	.000

## Data Availability

The data are not publicly available but are available upon reasonable request to the corresponding author (ptmdung@gmail.com).

## What is already known

- Nurses are critical in providing dementia care, yet in many countries nursing students may still graduate and enter the workforce with inadequate knowledge and skills in care provision.
- Several factors, including individual’s characteristics (age, gender, academic year, family history, living companions...) and experience in dementia care, have been found associated with their dementia knowledge and attitudes in many studies.
- Dementia care is increasingly a major health concern and demand for aging countries, like Vietnam, yet there is a lack of understanding of how well nursing students are prepared to meet the demand, and lack of comparison between three-year and four-year diploma nursing students - two training programs that are parallelly existent in medical training system in this nation.

## What this paper adds

- We found that Vietnamese nursing students were equipped with poor knowledge and given that they held quite positive attitude of dementia, their attitudes toward some items were relatively limited.
- We found a statistical difference in nursing students who attended three- and four-year training programs.
- The findings have important implications for the training programs to enhance the workforce’s readiness in meeting the increasing demand for dementia care.

## Abbreviations

AD: Alzheimer disease
ADKS: Alzheimer’s Disease Knowledge Scale
ADRD: Alzheimer disease and related disorders
DAS: Dementia Attitudes Scale
PWD: People with dementia
WHO: World Health Organization
